# The impact of parity on life course blood pressure trajectories: the HUNT study in Norway

**DOI:** 10.1007/s10654-018-0358-z

**Published:** 2018-01-24

**Authors:** Eirin B. Haug, Julie Horn, Amanda Rose Markovitz, Abigail Fraser, Corrie Macdonald-Wallis, Kate Tilling, Pål Richard Romundstad, Janet Wilson Rich-Edwards, Bjørn Olav Åsvold

**Affiliations:** 1 Department of Public Health and Nursing, Faculty of Medicine and Health Sciences, Norwegian University of Science and Technology, Postboks 8905, 7491 Trondheim, Norway; 2 The Harvard T.H Chan School of Public Health, Harvard University, Boston, MA USA; 3 MRC Integrative Epidemiology Unit and Population Health Sciences, University of Bristol, Bristol, UK; 4 Department of Obstetrics and Gynecology, Levanger Hospital, Nord-Trøndelag Hospital Trust, Levanger, Norway; 5 Connors Center for Women’s Health and Gender Biology, Brigham and Women’s Hospital, Harvard Medical Shcool, Boston, MA USA; 6 Department of Endocrinology, St. Olavs Hospital, Trondheim University Hospital, Trondheim, Norway

**Keywords:** Life course, Blood pressure, Parity, Pregnancy, Epidemiology

## Abstract

The drop in blood pressure during pregnancy may persist postpartum, but the impact of pregnancy on blood pressure across the life course is not known. In this study we examined blood pressure trajectories for women in the years preceding and following pregnancy and compared life course trajectories of blood pressure for parous and nulliparous women. We linked information on all women who participated in the population-based, longitudinal HUNT Study, Norway with pregnancy information from the Medical Birth Registry of Norway. A total of 23,438 women were included with up to 3 blood pressure measurements per woman. Blood pressure trajectories were compared using a mixed effects linear spline model. Before first pregnancy, women who later gave birth had similar mean blood pressure to women who never gave birth. Women who delivered experienced a drop after their first birth of − 3.32 mmHg (95% CI, − 3.93, − 2.71) and − 1.98 mmHg (95% CI, − 2.43, − 1.53) in systolic and diastolic blood pressure, respectively. Subsequent pregnancies were associated with smaller reductions. These pregnancy-related reductions in blood pressure led to persistent differences in mean blood pressure, and at age 50, parous women still had lower systolic (− 1.93 mmHg; 95% CI, − 3.33, − 0.53) and diastolic (− 1.36 mmHg; 95% CI, − 2.26, − 0.46) blood pressure compared to nulliparous women. The findings suggest that the first pregnancy and, to a lesser extent, successive pregnancies are associated with lasting and clinically relevant reductions in systolic and diastolic blood pressure.

**Electronic supplementary material:**

The online version of this article (10.1007/s10654-018-0358-z) contains supplementary material, which is available to authorized users.

## Introduction

Longitudinal studies have shown that blood pressure increases during a woman’s life [1–3]. In the first half of pregnancy blood pressure substantially decreases and then rises towards term [4–6]. Limited evidence from longitudinal studies following women from before to after first pregnancy suggests a woman’s first pregnancy is associated with a drop in blood pressure [6–8] that may persist for years postpartum [7]. The presence of a long-lasting drop in blood pressure after pregnancy has also been supported by some [9–12], but not all [13–15] studies that compared parous and nulliparous women at various time points after their first pregnancy. If long-lasting, this reduction in blood pressure may impact life course trajectories of blood pressure in parous women and reduce their cardiovascular disease (CVD) risk compared to men [16] and women who remain nulliparous [17, 18]. However, no study has followed women from pre-pregnancy to middle age to determine longitudinally whether the pregnancy-related drop in blood pressure persists into the age when CVD may emerge.

Using data from the population-based Nord-Trøndelag Health Study (the HUNT Study) linked with the Medical Birth Registry of Norway (MBRN) we examined blood pressure trajectories for women in the years preceding and following pregnancy and compared life course trajectories of blood pressure for parous and nulliparous women.

## Methods

### Study population

The HUNT Study is an ongoing longitudinal study in which all people aged 20 and above in Nord-Trøndelag county, Norway are invited to undergo an extensive health assessment, including clinical measurements and questionnaires [19]. So far three surveys have been conducted: HUNT1 (1984-86), HUNT2 (1995-97) and HUNT3 (2006-08). The population of Nord-Trøndelag is representative of Norway as a whole [20]. Participation rates for women were 89.9% in HUNT1 [21], 75.5% in HUNT2 [20] and 58.7% in HUNT3 [19].

**Fig. 1:**
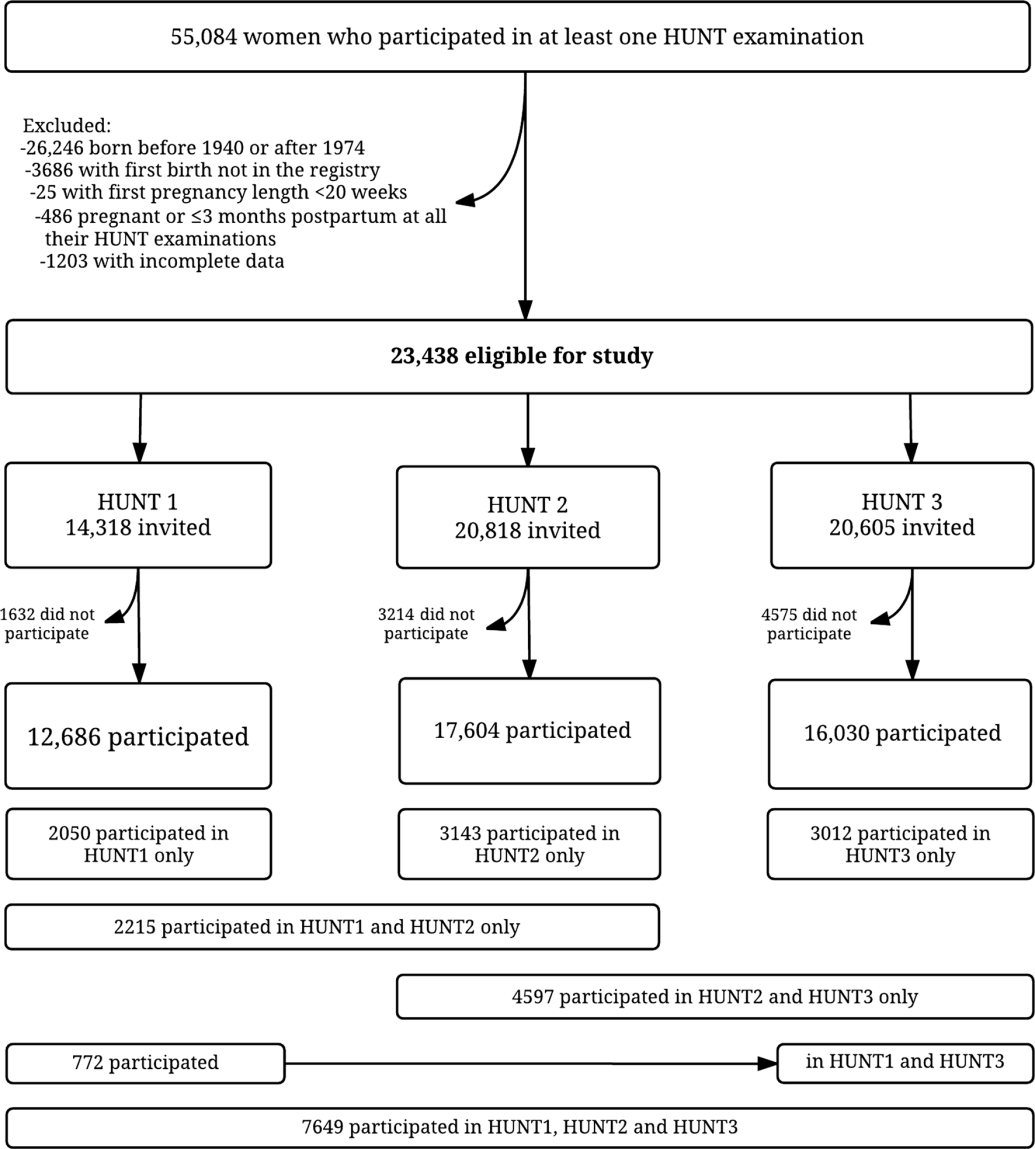
Flow chart of the study population

HUNT data were linked with the MBRN to retrieve information on births using the unique personal identification numbers assigned to Norwegians at birth or immigration. All births in Norway since 1967 have been recorded in the MBRN [22], and data were available through 2012. Among 55,084 women who had taken part in at least one HUNT survey, we excluded 26,246 women who were born before 1940 or after 1974 since their complete reproductive history may not have been captured between 1967 and 2012. Among the remaining 28,838 women, 5400 (18.7%) were excluded for the following reasons: We excluded 3686 women who did not have their first birth registered in the MBRN and 25 women whose first recorded pregnancy was shorter than 20 weeks since it was uncertain whether these shorter pregnancies would cause lasting cardiovascular changes. Finally, we excluded 486 women whose only blood pressure measurements were performed in pregnancy or up to 3 months postpartum and 1203 women with incomplete information on blood pressure, smoking or education, leaving 23,438 women for analysis (Fig. 1). Descriptive characteristics of excluded versus included women are shown in Supplemental Table 1.

### Blood pressure and covariates

In each HUNT survey, blood pressure was measured by trained staff after the person had rested. In HUNT1 [21] blood pressure was measured manually two times with a 1-min interval using a sphygmomanometer, and in HUNT2 [20] and HUNT3 [19] blood pressure was measured three times with 1-min intervals using an automatic oscillometric method (Dinamap, Critikon, Florida) with cuff size adjusted to arm circumference. We used the means of the 1st and 2nd (HUNT1) or 2nd and 3rd (HUNT2 and HUNT3) measurements in the analyses. In HUNT3, due to sick leave amongst staff, 2016 women did not have their 3rd blood pressure measurement taken, and for them we used the 2nd measurement. To account for bias due to use of antihypertensive medication, blood pressure measurements from women using antihypertensives were, according to recommendations by Cui et al. [23] and Tobin et al. [24], amended by adding 10 and 5 mmHg to the measured systolic and diastolic blood pressure, respectively. We excluded blood pressure measurements performed in pregnancy or within 3 months postpartum.

Body mass index (BMI; weight in kg divided by the squared height in m^2^) was measured at each HUNT examination. The HUNT questionnaires included information on smoking and anti-hypertensive medication (all HUNT surveys), use of oral contraceptives and breastfeeding duration (HUNT2 and HUNT3), and highest obtained educational level (HUNT1 and HUNT2); lower secondary (up to 9 years), upper secondary (10–12 years) and tertiary education (college or university). Information on work titles (HUNT3) was obtained from a structured interview. Due to lack of educational information for women who participated only in HUNT3, we derived educational status from work titles for 4041 women based on recommendations from Statistics Norway [25].

Information on hypertensive disorders in pregnancy (preeclampsia, gestational hypertension, and pre-pregnancy chronic hypertension) was retrieved from the MBRN, which records these disorders from standardized forms filled in at the birth clinics and returned shortly after delivery. Validation studies within the HUNT population have shown that 88% of preeclampsia cases in the MBRN were confirmed by evidence in hospital records [26], and 74% of cases of gestational hypertension in the MBRN had evidence of gestational hypertension or preeclampsia in hospital records [27].

### Statistical analysis

We used a linear spline mixed effects model [28] to estimate blood pressure trajectories for women who remained nulliparous or became parous at some point during 1967–2012, defined as having at least one pregnancy lasting beyond 20 weeks of gestation. To account for repeated observations (up to three per woman) and reflect the heterogeneity in the data, all models included a random intercept and a random slope. The effect of pregnancy was modeled using two variables: The first indicated whether the measurement occurred pre- versus post-pregnancy and provided an estimate of the immediate change in blood pressure following pregnancy, and the other indicated continuous time post pregnancy and gave an estimate of the change in blood pressure slope after pregnancy. Using linear splines allowed the change in blood pressure to vary by age interval, enabling non-linear trends in average blood pressure with age to be modeled. Knots (points at which the linear slope changed) were selected using the Bayesian Information Criterion (BIC) [29] to compare multivariable models with different sets of knots (age intervals of 2, 4, 5, 6, 8, and 10 years). Knots were placed at 10-year age intervals as models with more knots did not prove superior. We included interaction terms to allow the age-dependent changes in blood pressure to vary between parous and nulliparous women, and to allow the effects of age and pregnancy on blood pressure to vary by levels of covariates. The estimates were adjusted for age, HUNT survey, education (as a proxy for socioeconomic status) and ever daily smoking. Blood pressure measurements up to 68 years of age were included, but blood pressure trajectories were presented for the age range 20–60 years due to limited data from older women. Predicted blood pressure trajectories are displayed for representative nulliparous women and parous women with first birth at 23, second at 27 and third at 30 years of age, corresponding to the median ages at births in our study population. In an analogous approach, we used logistic regression analysis to estimate trajectories of the prevalence of hypertension, defined as self-reported use of antihypertensives or blood pressure ≥ 140 mmHg systolic or ≥ 90 mmHg diastolic.

In analyses restricted to parous women, we examined whether the effect of pregnancy on blood pressure varied by age at first pregnancy. To confirm that the average blood pressure trajectories drawn using data from all examinations among all women were representative of within-woman changes in blood pressure across time, we performed a sensitivity analysis excluding women who had only one blood pressure measurement. Further to confirm that the trajectories represented the actual within-woman change in blood pressure due to pregnancy, we studied the difference in blood pressure change for women who had their pregnancy between HUNT2 and HUNT3 to women who remained nulliparous throughout the same interval and were 43 years or younger at HUNT2, the maximum age at HUNT2 of those who went on to have their first birth. To examine the extent of confounding by oral contraceptive use and BMI, the analysis of change in blood pressure between HUNT2 and HUNT3 was adjusted for change in BMI and oral contraceptive use between the HUNT surveys. Also, in order to investigate the potential mediating effect of breastfeeding upon the association between pregnancy and a drop in blood pressure, we also categorized women who delivered according to breastfeeding duration after first pregnancy. Lastly, we estimated the blood pressure trajectories for women with a hypertensive disorder and normotension in first pregnancy. All statistical analyses were carried out using Stata IC 13 (StataCorp, College Station, Texas) and MLwiN [30] version 2.34.

## Results

Characteristics of the 21,513 parous and 1925 nulliparous women included in the analysis are given in Table 1. Compared to parous women, nulliparous women were more likely to be obese, but were less likely to report ever smoking or ever use of oral contraceptives. In total 46,320 blood pressure measurements were taken, 3417 from nulliparous and 42,903 from parous women, and of the latter, 2963 were collected pre-pregnancy and 39,940 post-pregnancy. A total of 7649 (33%) women participated in all three HUNT surveys and therefore had their blood pressure measured on three occasions, 7584 (33%) in two and 8199 (34%) in only one HUNT survey (Fig. 1). The distribution of blood pressure measurements by age group and HUNT survey is shown in Supplemental Figure 1. Median ages were 23 years at first birth, 27 at second and 30 at the third birth. Blood pressure measurements in HUNT covered time periods spanning from 20 years before to 40 years after the first pregnancy.

**Table 1:** Descriptive characteristics of the study population

Characteristics	Nulliparous (n = 1925)	Parous (n = 21,513)
Birthyear, median (IQR)	1958 (1949–1966)	1958 (1951–1965)
Ever smoked daily, n (%)		
No	924 (48)	8500 (40)
Yes	1001 (52)	13,013 (60)
Education, n (%)		
Lower secondary	437 (23)	3823 (18)
Upper secondary	814 (42)	10,061 (47)
Tertiary	674 (35)	7629 (35)
Ever used oral contraceptives, n (%)*		
No	693 (36)	4380 (20)
Yes	708 (37)	13,077 (61)
Missing	524 (27)	4056 (19)
Ever used blood pressure medication, n (%)		
No	1721 (89)	19,075 (89)
Yes	204 (11)	2434 (11)
Missing	0 (0)	4 (0)
Births, n (%)		
1	N/A	2577 (12)
2	N/A	9778 (46)
3 or more	N/A	9158 (42)
Age at 1st birth, median (IQR)	N/A	23 (20–26)
Year of 1st birth, median (IQR)	N/A	1981 (1973–1990)
Breastfeeding length of first child, n (%)*		
No breastfeeding	N/A	994 (5)
< 3 months	N/A	2864 (13)
3–6 months	N/A	5437 (25)
> 6 months	N/A	7401 (34)
Missing	N/A	4817 (22)
No. of HUNT exams, n (%)		
1	898 (47)	7307 (34)
2	562 (29)	7022 (33)
3	465 (24)	7184 (33)
*Time varying covariates*		
Number of observations, n (%)	3417 (7)	42,903 (93)
BMI at HUNT exam, kg/m^2^		
< 25	1792 (52)	24,022 (56)
25–29.9	953 (28)	12,935 (30)
≥ 30	648 (19)	5881 (14)
Missing	24 (1)	65 (0.2)
Current use of oral contraceptives, n (%)*		
No	1684 (49)	22,686 (53)
Yes	234 (7)	2797 (7)
Missing	1499 (44)	17,420 (41)
Current use of blood pressure medication, n (%)		
No	3192 (93)	40,461 (94)
Yes	219 (6)	2338 (6)
Missing	6 (0.2)	104 (0.2)

*Queried at HUNT2 and HUNT3

**Fig. 2:**
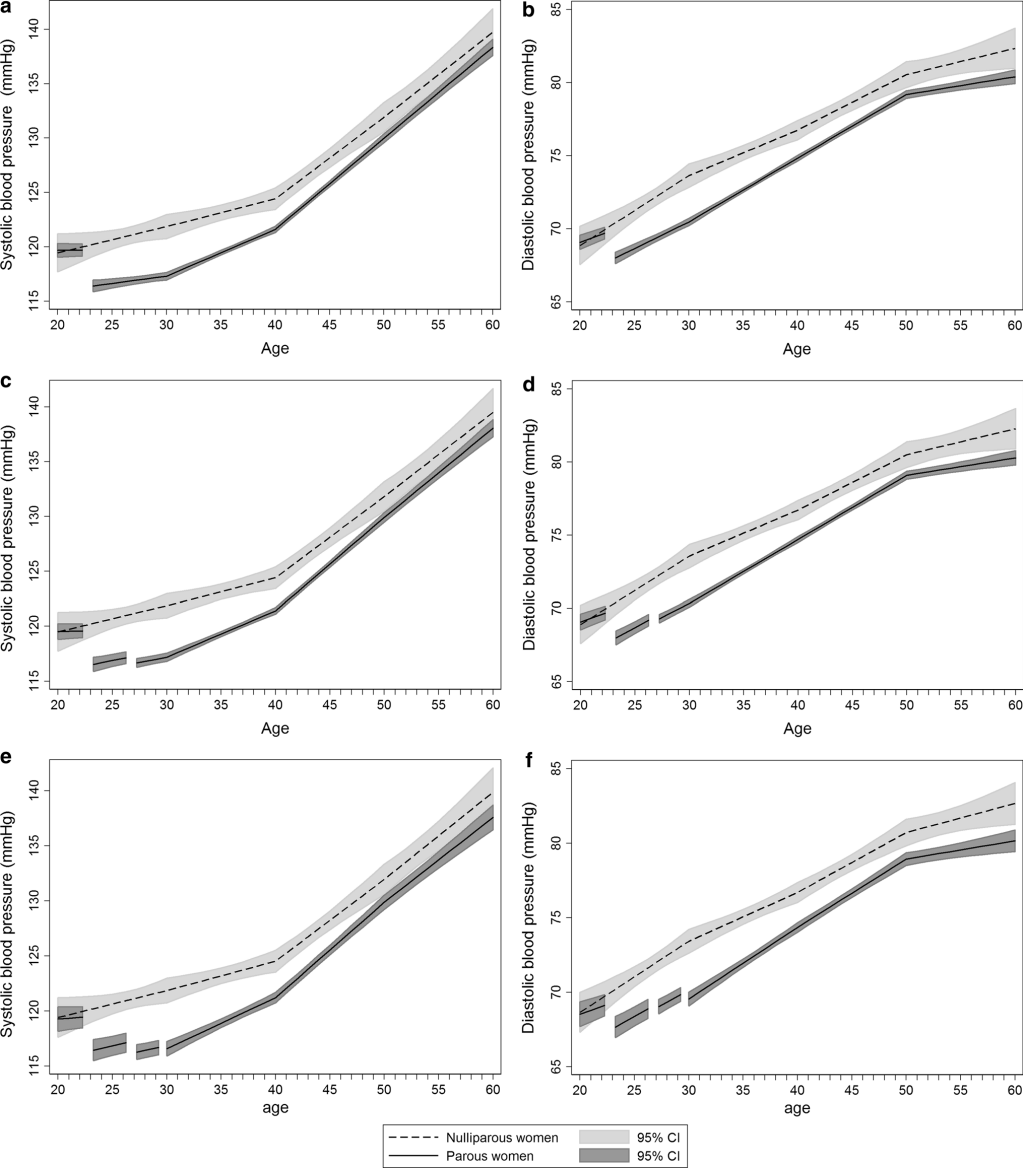
Mean systolic and diastolic blood pressure life course trajectories for nulliparous and parous women with one or more births (**a** and **b**), two or more births (**c** and **d**) and three or more births (**e** and **f**). Trajectories are drawn for women with covariates fixed at their means and with gaps in the graph of parous women corresponding to pregnancy and 3-month postpartum periods with the 1st birth at age 23, 2nd at 27 and 3rd at 30 years. Estimates are adjusted for age, HUNT survey, education and ever daily smoking

Figure 2 shows trajectories of systolic and diastolic blood pressure for parous and nulliparous women for the age interval 20–60 years. Women who became parous by the end of follow up and nulliparous women had indistinguishable mean blood pressure levels at age 20 (when both groups were nulliparous) until the first birth of the parous women, after which the blood pressures of the newly parous women fell abruptly (Fig. 2a, b). The mean adjusted changes in systolic and diastolic blood pressure from pre to post first pregnancy were − 3.32 mmHg (95% CI, − 3.93, − 2.71) and − 1.98 mmHg (95% CI, − 2.43, − 1.53), respectively (Table 2). Second and third pregnancies were also associated with blood pressure declines, though smaller than those seen in the first pregnancy (Fig. 2c–f, Table 2).

**Table 2:** Estimated mean change in systolic and diastolic blood pressure from pre- to post-pregnancy among parous women

	Pregnancy one^a^			Pregnancy two^b^			Pregnancy three^c^		
	Blood pressure change	95% CI	*p* value	Blood pressure change	95% CI	*p* value	Blood pressure change	95% CI	*p* value
Systolic (mmHg)									
Model 1^d^	− 3.42	[− 3.98, − 2.85]	< 0.001	− 0.68	[− 1.28, − 0.07]	0.028	− 0.22	[0.97, 0.53]	0.563
Model 2^e^	− 3.32	[− 3.93, − 2.71]	< 0.001	− 0.68	[− 1.30, − 0.06]	0.031	− 0.24	[− 1.00, 0.52]	0.537
Diastolic (mmHg)									
Model 1^d^	− 2.00	[− 2.42, − 1.59]	< 0.001	− 0.33	[− 0.77, 0.11]	0.138	− 0.62	[− 1.15, − 0.10]	0.021
Model 2^e^	− 1.98	[− 2.43, − 1.53]	< 0.001	− 0.31	[− 0.75, 0.14]	0.182	− 0.59	[− 1.13, − 0.06]	0.031

^a^Estimates are obtained from the trajectory models depicted in Fig. 2a and b where nulliparous women and all women with one or more children are included (n = 23,168)

^b^Estimates are obtained from the trajectory models depicted in Fig. 2c and d where nulliparous women and all women with two or more children are included (n = 20,861)

^c^Estimates are obtained from the trajectory models depicted in Fig. 2e and f where nulliparous women and all women with three or more children are included (n = 11,083)

^d^Estimates are adjusted for age and HUNT survey

^e^Estimates are adjusted for age, HUNT survey, education and ever daily smoking

It took parous women roughly a decade to reach their mean pre-pregnancy blood pressure levels. From age 30 to 40 years, parous women had a faster rise in blood pressure compared with nulliparous women (Supplemental Table 2). Yet, the lower blood pressure in parous compared with nulliparous women lasted beyond 50 years of age (Fig. 2). Compared with nulliparous women, systolic blood pressure of parous women differed by − 1.93 mmHg (95% CI, − 3.33, − 0.53) at age 50 and − 1.38 mmHg (95% CI, − 3.56, 0.80) at age 60, while diastolic blood pressure differed by − 1.36 mmHg (95% CI, − 2.26, − 0.46) at age 50 and − 1.95 mmHg (95% CI, − 3.34, − 0.55) at age 60 (Supplemental Table 3).

Prior to pregnancy, the prevalence of hypertension was lower among future parous compared with never parous women. The prevalence among parous women declined after pregnancy, leading to a long-lasting greater difference in prevalence between parous and nullparous women that attenuated from 40 to 50 years of age (Supplemental Figure 2).

We examined whether the effect of pregnancy on blood pressure varied by age at first pregnancy. The blood pressure decline from pre to post first pregnancy was only slightly smaller (− 0.03 mmHg; 95% CI, − 0.18, 0.12) for systolic and slightly larger (0.06 mmHg; 95% CI, − 0.05, 0.17) for diastolic for each 1-year higher age at first pregnancy. When restricting our analysis to the 15,233 women with repeated (2 or 3) blood pressure measurements we observed similar trajectories as in our main analysis (Supplemental Figure 3), confirming that our main results were representative of within-woman changes in blood pressure. As a sensitivity analysis, we examined how the amendment for the effect of antihypertensive medication influenced our results and found that the shape of the trajectories remained essentially unchanged when we used the original, unamended blood pressure values in the analysis (Supplemental Figure 4).

Our analysis of within-woman change in blood pressure comparing the 621 women who gave birth to their first child between HUNT2 and HUNT3 to the 427 who remained nulliparous confirmed that pregnancy was associated with reductions in systolic and diastolic blood pressure similar to those observed in the main analysis (Supplemental Table 4); the estimated mean drop after pregnancy was − 3.99 mmHg (95% CI, − 5.98, − 1.99) for systolic and − 3.04 mmHg (95% CI, − 4.43, − 1.64) for diastolic blood pressure. Additional adjustment for oral contraceptive use and BMI did not substantially attenuate the estimated association between pregnancy and blood pressure change (Supplemental Table 5). The blood pressure change was broadly similar across categories of breastfeeding duration; however, 79% of women with first birth between HUNT2 and HUNT3 breastfed for > 6 months after their first pregnancy, and the low number of women with no or short breastfeeding duration prevented precise estimates for those groups (Supplemental Table 6).

Among 21,513 parous women, 20,038 had normotension and 1475 had a hypertensive disorder in first pregnancy (preeclampsia, 994; gestational hypertension, 433; pre-pregnancy chronic hypertension, 48). There was some evidence that the blood pressure drop from pre to post first pregnancy differed between the two groups (P_interaction_ = 0.195 for systolic and 0.007 for diastolic blood pressure). In women with normotension in first pregnancy, the mean adjusted changes from pre to post first pregnancy were − 3.43 mmHg (95% CI, − 4.05, − 2.80) in systolic and − 2.15 mmHg (95% CI, − 2.62, − 1.69) in diastolic blood pressure. In women with a hypertensive disorder in first pregnancy, the corresponding changes were − 2.02 mmHg (95% CI, − 4.08, 0.04) systolic, but only 0.01 mmHg (95% CI, − 1.50, 1.51) diastolic. Women with a hypertensive disorder in first pregnancy had higher mean blood pressure throughout the age span, compared with both nulliparous women and women with a normotensive first pregnancy (Supplemental Figure 5).

## Discussion

This study provides evidence that systolic and diastolic blood pressure drop after a woman’s first birth and suggests that pregnancy itself induces differences in blood pressure between parous women post-pregnancy and nulliparous women. Our results also show that it takes approximately a decade for parous women to reach the levels they experienced pre-pregnancy, and they do not reach the levels of nulliparous women until beyond menopause.

Our study is the first to include blood pressure measurements spanning from pre-pregnancy up to 40 years postpartum and is the first to examine blood pressure trajectories across a woman’s life course taking into account the timing of pregnancy. The magnitude of drop in blood pressure associated with a woman’s first pregnancy of − 3 mmHg systolic and − 2 mmHg diastolic is consistent with previous studies that examined changes in blood pressure from pre-pregnancy to postpartum [6–8]. In the longitudinal Cardia study of 2304 women, systolic and diastolic blood pressure dropped by − 2 mmHg over an interval of 2–20 years for women who had a first birth during the interval [7]. Similar differences between parous and nulliparous women were seen at age 36, but had disappeared by age 53 in a British cohort study of 2977 women [12]. In a Swiss cohort study [9], parity was associated with lower blood pressure before 60 years, but with a higher blood pressure after 60 years of age. Other cross-sectional studies examining blood pressure or risk of hypertension by parity status have reported either no significant association [13–15] or lower blood pressure among parous women [10, 11], with stronger association seen in premenopausal women [10, 11].

Our large study size, almost ten-fold more women than previous individual longitudinal studies, yielded precise blood pressure estimates. A major advantage of our study is that in addition to comparing parous women to women who remained nulliparous throughout their life, we were also able to compare pre- and post-pregnancy blood pressure among parous women. Most previous studies only compared parous to nulliparous women to estimate the long-term effect of pregnancy on blood pressure. This approach is susceptible to confounding by socioeconomic and behavioral factors and by health conditions such as polycystic ovary syndrome that impact fertility and may also affect blood pressure [31, 32]. In our data, the lack of difference in mean blood pressure in early adulthood between future parous and never parous women, the abrupt drop in blood pressure trajectory at the time of pregnancy, and the within-woman drop in blood pressure from pre to post first pregnancy all suggest that effects of parity explain most of the difference in blood pressure between parous and nulliparous women. Nonetheless, the higher prevalence of hypertension in early adulthood among never parous compared with future parous women suggests that early-onset factors influencing parity may also contribute to higher blood pressure in nulliparous women.

We used a mixed effects model [33] to account for correlated repeated measures of blood pressure in the same woman and model the subject variation in blood pressure levels and slopes between women. This allowed us to estimate within-woman blood pressure trajectories, avoiding the pitfalls of using purely cross-sectional information which may not correctly represent within-subject change over time. Two thirds of the study subjects participated in more than one HUNT exam and we obtained similar results when restricting to this exclusively longitudinal subgroup. The method for blood pressure measurement in HUNT1 differed from that in HUNT2 and HUNT3; therefore, we adjusted for HUNT survey in the analyses. Also, the pregnancy-related drop in blood pressure was confirmed when we examined within-woman change in blood pressure between HUNT2 and HUNT3 and found that women giving birth in this interval experienced drops in systolic and diastolic blood pressure comparable to the ones found in our main analysis.

In our main analysis, we controlled for age, education and smoking. Unfortunately, we were unable to adjust for pre-pregnancy BMI and oral contraceptive use, as these covariates were lacking for the majority of participants. However, in the analysis of within-woman change in blood pressure, adjustment for BMI and oral contraceptive use did not markedly attenuate the estimates, indicating that the lack of adjustment for these variables is not a source of substantial bias in the main analysis. We cannot exclude residual confounding due to other factors related to both parity and later blood pressure levels, for example infertility-associated health conditions. However, these factors are unlikely to explain the within-woman drop in blood pressure at the time of pregnancy. Non-participation in HUNT was related to age, socioeconomic factors and adverse health outcomes, including a higher prevalence of cardiovascular disease and diabetes, but not to use of antihypertensive medication [34] and we do not expect non-participation to have affected the shape of or differences between the trajectories.

There was a secular decrease in blood pressure between HUNT2 and HUNT3 [35], as also observed in other populations [36] over the same time period and this may be due to dietary changes and increased use of antihypertensive medication. Although we did add constants to the measured blood pressure values of individuals treated for hypertension, as recommended [23, 24], the slope in blood pressure with age may be underestimated. However, we have no reason to believe that this underestimate would substantially affect nulliparous differently from parous individuals and alter our overall findings. While the study population is fairly representative of the population of Norway [20], it is an ethnically homogenous population which may limit the generalizability of these findings. There is some evidence that the effect of pregnancy on blood pressure may be weaker for Black compared with White women [7]. It is also possible that the effect of pregnancy on blood pressure may differ by pregnancy characteristics. In our study, the drop in diastolic pressure from pre to post pregnancy was absent among women with hypertensive pregnancy disorders, but their drop in systolic blood pressure did not convincingly differ from that observed in women with normotensive pregnancies.

One possible explanation for the longlasting differences in blood pressure between parous and nulliparous women is that changes in vascular function that occur in response to pregnancy persist postpartum. There are a number of cardiovascular adaptations to pregnancy that increase blood flow to organs, including a large increase in cardiac output and a corresponding decrease in vascular resistance [37]. Some of these adaptations, such as increased heart rate, appear to normalize quickly [6] while others such as reduced vascular resistance [6] and increased arterial compliance [8] appear to last at least 1 year postpartum. The decrease in vascular resistance following pregnancy at 1 year postpartum [6] may partly be explained by reduced arterial stiffness [8] which also was found to be present at 1 year postpartum. Pregnancy may impart lasting changes to cardiovascular structure and function in a similar manner to regular exercise [38].

Alternatively, other factors that accompany pregnancy may contribute to the lower post-pregnancy blood pressure. In two cross-sectional studies [39, 40], one of which was conducted within the HUNT study cohort [39], longer duration of breastfeeding was associated with lower blood pressure among parous women. Those results may suggest that breastfeeding mediates the association between parity and blood pressure, but could also have arisen due to higher pre-pregnancy blood pressure in women with short or no breastfeeding. In our longitudinal analysis, we saw no dose–response relationship between breastfeeding duration and blood pressure change from pre to post first pregnancy. Although our longitudinal sample was too small to make conclusive inferences, our results suggest breastfeeding does not mediate the drop in blood pressure observed after pregnancy. It is also possible that lifestyle changes post-pregnancy contribute to decreasing blood pressure. This would be consistent with findings from a British cohort that both women and men had lower blood pressure if they had one or more children compared with none, with little difference in magnitude by sex [12]. There is a small, lasting weight gain (mean, 0.5–3 kg) [41] associated with pregnancy; this would expectedly contribute to a higher blood pressure. In our data, adjustment for pre- to post-pregnancy change in BMI slightly attenuated the estimates.

A 2–3 mmHg lower blood pressure lasting from first pregnancy to beyond 50 years of age is likely to have a significant influence on risk of CVD, as even a 2 mmHg reduction in diastolic blood pressure was found to reduce the risk of coronary heart disease by 6% and the risk of stroke and transient ischemic attacks by 15% [42]. The pregnancy-related drop in blood pressure may contribute to the lower CVD risk observed in women compared with men at younger age. It has been estimated that sex differences in blood pressure may explain 20% of the sex difference in CVD mortality below 50 years of age, but little or no of the sex difference at older ages [16]. Finally, the pregnancy-related drop in blood pressure provides a possible explanation why the risk of pre-eclampsia is higher in the first compared with the second pregnancy since higher pre-pregnancy blood pressure is associated with increased risk of pre-eclampsia. The risk of pre-eclampsia is more than halved from the first to subsequent pregnancies [43] and this reduced risk is present for interpregnancy intervals up to approximately 10 years [43]. Our results are consistent with the hypothesis that lower blood pressure following a first pregnancy reduces the risk of preeclampsia and that this protective effect gradually diminishes but can remain for up to a decade [44, 45], at which time mean blood pressure approached its pre-pregnancy level in our data.

## Conclusion

A woman’s first pregnancy and to a lesser extent her subsequent pregnancies, are associated with reductions in systolic and diastolic blood pressure that persist over decades. The decreases in blood pressure resulting from pregnancies may provide a protective effect against hypertension and CVD. Our results may help explain CVD risk differences defined by parity and sex and why the risk of preeclampsia is higher in the first compared with later pregnancies.

## Electronic supplementary material

Below is the link to the electronic supplementary material.

Supplementary material 1 (DOCX 456 kb)
