# Correction to: The impact of parity on life course blood pressure trajectories: the HUNT study in Norway

**DOI:** 10.1007/s10654-018-0412-x

**Published:** 2018-07-06

**Authors:** Eirin B. Haug, Julie Horn, Amanda Rose Markovitz, Abigail Fraser, Corrie Macdonald-Wallis, Kate Tilling, Pål Richard Romundstad, Janet Wilson Rich-Edwards, Bjørn Olav Åsvold

**Affiliations:** 1 Department of Public Health and Nursing, Faculty of Medicine and Health Sciences, Norwegian University of Science and Technology, Postboks 8905, 7491 Trondheim, Norway; 2 The Harvard T.H Chan School of Public Health, Harvard University, Boston, MA USA; 3 MRC Integrative Epidemiology Unit and Population Health Sciences, University of Bristol, Bristol, UK; 4 Department of Obstetrics and Gynecology, Levanger Hospital, Nord-Trøndelag Hospital Trust, Levanger, Norway; 5 Connors Center for Women’s Health and Gender Biology, Brigham and Women’s Hospital, Harvard Medical School, Boston, MA USA; 6 Department of Endocrinology, St. Olavs Hospital, Trondheim University Hospital, Trondheim, Norway

## Correction to: European Journal of Epidemiology 10.1007/s10654-018-0358-z

The article “The impact of parity on life course blood pressure trajectories: the HUNT study in Norway”, written by Eirin B. Haug, Julie Horn, Amanda Rose Markovitz, Abigail Fraser, Corrie Macdonald-Wallis, Kate Tilling, Pål Richard Romundstad, Janet Wilson Rich-Edwards and Bjørn Olav Åsvold, was originally published electronically on the publisher’s internet portal (currently SpringerLink) on 24 January 2018 without open access.

With the author(s)’ decision to opt for Open Choice the copyright of the article changed on 30 May 2018 to © The Author(s) 2018 and the article is forthwith distributed under the terms of the Creative Commons Attribution 4.0 International License (http://creativecommons.org/licenses/by/4.0/), which permits use, duplication, adaptation, distribution and reproduction in any medium or format, as long as you give appropriate credit to the original author(s) and the source, provide a link to the Creative Commons license and indicate if changes were made.

