# Supplementary material for: The impact of parity on life course blood pressure trajectories: the HUNT study in Norway
Source: Eur J Epidemiol. 2018 Jan 24;33(8):751–61. doi: 10.1007/s10654-018-0358-z (PMC6061132; doi:10.1007/s10654-018-0358-z)
Supplement: Supplementary file 1 — Supplementary material 1 (DOCX 456 kb) [file 10654_2018_358_MOESM1_ESM.docx]

| Supplementary material |
| --- |
| The impact of parity on life course blood pressure trajectories. The HUNT Study in Norway. |

| **Supplemental Table 1. Descriptive characteristics of female HUNT participants born in the eligible birth cohorts 1940-1974, by inclusion status** | | | | | |
| --- | --- | --- | --- | --- | --- |
| **Characteristics** | | | **Included in the analyses**  (n=23,438) | | **Excluded from the analyses**  (n=5400) |
| Birthyear, median (IQR) | | | 1958 (1951 – 1965) | | 1945 (1942 - 1956) |
| Age at last HUNT participation,  median (IQR) | | | | 45 (37 - 54) | 53 (35 - 64) |
| Ever smoked daily, n  (% of non-missing) | | |  | |  |
|  |  | No | 9424 (40) | | 1582 (37) |
|  |  | Yes | 14,014 (60) | | 2658 (63) |
|  | Missing, n (%) | | 0 | | 1160 (22) |
| Education, n  (% of non-missing) | | |  | |  |
|  |  | Lower Secondary | 4260 (18) | | 1744 (43) |
|  |  | Upper Secondary | 10875 (46) | | 1481 (37) |
|  |  | Tertiary | 8303 (35) | | 831 (21) |
|  | Missing, n (%) | | 0 | | 1344 (25) |
| Ever used oral contraceptives, n  (% of non-missing)^*^ | | |  | |  |
|  |  | No | 5073 (27) | | 1680 (52) |
|  |  | Yes | 13785 (73) | | 1572 (48) |
|  | Missing, n (%) | | 4580 (20) | | 2148 (40) |
| Ever used blood pressure medication, n  (% of non-missing) | | |  | |  |
|  |  | No | 20,796 (89) | | 4391 (81) |
|  |  | Yes | 2638 (11) | | 1004 (19) |
|  | Missing, n (%) | | 4 (0.02) | | 5 (0.1) |
| Parity, n (%) | | |  | |  |
|  | Nulliparous | | 1925 (8) | | 269 (5) |
|  | Parous | | 21,513 (92) | | 5131 (95) |
|  |  | 1 birth | 2362 (10) | | 409 (8) |
|  |  | 2 births | 9500 (41) | | 1666 (31) |
|  |  | 3 or more births | 9651 (41) | | 3053 (57) |
|  |  | unknown number of births | 0 | | 3 (0.1) |
| Age at 1^st^ birth, median (IQR)^**^ | | | 23 (20 - 26) | | 21 (19 - 23) |
|  | Missing, n (%) | | 0 | | 1162 (22) |
| Year of 1^st^ birth, median (IQR)^**^ | | | 1981 (1973–1990) | | 1966 (1964 - 1980) |
|  | Missing, n (%) | | 0 | | 1. (29) |
| ^*^ Queried at HUNT2 and HUNT3  ^**^ For women whose first birth was prior to the inception of Medical Birth Registry of Norway in 1967, information on age and year of first birth is based on the women’s report at participation in HUNT | | | | | |

| **Supplemental Table 2. Predicted increase per year in systolic and diastolic pressure by age at follow-up in parous and nulliparous women.** | | | | | | | | | | |
| --- | --- | --- | --- | --- | --- | --- | --- | --- | --- | --- |
|  |  | **Nulliparous** | |  | **Parous^*^** | |  |  | **Difference** |  |
| **Age interval** |  | Blood pressure^†^ | 95% CI |  | Blood pressure^†^ | 95% CI |  | Blood pressure^†^ | 95% CI | p-value |
| **Systolic** (mmHg/year) |  |  |  |  |  |  |  |  |  |  |
| 20‒23 years |  | 0.242 | [0.003 , 0.480] |  | 0.012 | [-0.076 , 0.100] |  | -0.230 | [-0.483 , 0.023] | 0.075 |
| 24‒30 years |  | 0.242 | [0.003 , 0.480] |  | 0.134 | [0.047 , 0.220] |  | -0.108 | [-0.361 , 0.144] | 0.401 |
| 30‒40 years |  | 0.255 | [0.098 , 0.412] |  | 0.430 | [0.383 , 0.476] |  | 0.174 | [0.012 , 0.337] | 0.035 |
| 40‒50 years |  | 0.751 | [0.584 , 0.918] |  | 0.839 | [0.792 , 0.886] |  | 0.089 | [-0.083 , 0.260] | 0.310 |
| 50‒60 years |  | 0.781 | [0.535 , 1.028] |  | 0.836 | [0.765 , 0.908] |  | 0.055 | [-0.198 , 0.308] | 0.671 |
|  |  |  |  |  |  |  |  |  |  |  |
| **Diastolic** (mmHg/year) |  |  |  |  |  |  |  |  |  |  |
| 20‒23 years |  | 0.478 | [0.304 , 0.651] |  | 0.280 | [0.216 , 0.344] |  | -0.198 | [-0.382 , -0.014] | 0.035 |
| 24‒30 years |  | 0.478 | [0.304 , 0.651] |  | 0.364 | [0.302 , 0.427] |  | -0.114 | [-0.297 , 0.070] | 0.226 |
| 30‒40 years |  | 0.312 | [0.204 , 0.421] |  | 0.437 | [0.405 , 0.469] |  | 0.125 | [0.013 , 0.237] | 0.029 |
| 40‒50 years |  | 0.379 | [0.268 , 0.490] |  | 0.435 | [0.404 , 0.467] |  | 0.056 | [-0.058 , 0.170] | 0.335 |
| 50‒60 years |  | 0.181 | [0.017 , 0.344] |  | 0.122 | [0.075 , 0.168] |  | -0.059 | [-0.226 , 0.109] | 0.491 |
| ^*^ Predicted for parous women having their first birth at age 23, corresponding to median age at first birth in our study population.  ^†^ Estimates are based on the trajectory models depicted in Figure 2a and 2b and adjusted for age, HUNT survey, education and ever daily smoking. | | | | | | | | | | |

| **Supplemental Table 3. Predicted mean systolic and diastolic blood pressure by age at follow-up in parous and nulliparous women.** | | | | | | | | | | | | | | | | |  |
| --- | --- | --- | --- | --- | --- | --- | --- | --- | --- | --- | --- | --- | --- | --- | --- | --- | --- |
|  | |  | Nulliparous | | | |  | | | Parous^*^ | | |  | | Difference | | |
|  | |  | Blood pressure^†^ | | 95% CI | | |  | | Blood pressure^†^ | 95% CI | |  | | Blood pressure^†^ | 95% CI | p-value |
| Systolic (mmHg) |  | | |  |  | |  | | |  |  | |  | |  |  |  |
| 20 years | |  | 119.44 | | [117.69 , 121.19] | |  | | | 119.68 | [119.03 , 120.33] | |  | | 0.24 | [-1.58 , 2.05] | 0.797 |
| 1^st^ birth in parous women occurs at age 23 | | | | | | | | | | | | | | | | | |
| 30 years | |  | 121.86 | | [120.75 , 122.97] | | | |  | 117.30 | [116.95 , 117.65] | | |  | -4.56 | [-5.70 , -3.42] | <0.001 |
| 40 years | |  | 124.41 | | [123.43 , 125.40] | | | |  | 121.60 | [121.32 , 121.88] | | |  | -2.82 | [-3.84 , -1.79] | <0.001 |
| 50 years | |  | 131.92 | | [130.56 , 133.28] | | | |  | 129.99 | [129.58 , 130.40] | | |  | -1.93 | [-3.33 , -0.53] | 0.007 |
| 60 years | |  | 139.73 | | [137.59 , 141.88] | | | |  | 138.35 | [137.62 , 139.09] | | |  | -1.38 | [-3.56 , 0.80] | 0.215 |
|  | |  |  | |  | | | |  |  |  | | |  |  |  |  |
| Diastolic (mmHg) | |  |  | |  | | | |  |  |  | | |  |  |  |  |
| 20 years | |  | 68.85 | | [67.54 , 70.17] | | | |  | 69.07 | [68.59 , 69.56] | | |  | 0.22 | [-1.14 , 1.58] | 0.750 |
| 1^st^ birth in parous women occurs at age 23 | | | | | | | | | | | | | | | | | |
| 30 years | |  | 73.63 | | [72.83 , 74.43] |  | | | | 70.46 | [70.21 , 70.71] |  | | | -3.17 | [-3.99 , -2.35] | <0.001 |
| 40 years | |  | 76.75 | | [76.09 , 77.41] |  | | | | 74.83 | [74.64 , 75.02] |  | | | -1.92 | [-2.61 , -1.23] | <0.001 |
| 50 years | |  | 80.54 | | [79.66 , 81.42] |  | | | | 79.18 | [78.92 , 79.45] |  | | | -1.36 | [-2.26 , -0.46] | 0.003 |
| 60 years | |  | 82.35 | | [80.98 , 83.72] |  | | | | 80.40 | [79.94 , 80.87] |  | | | -1.95 | [-3.34 , -0.55] | 0.006 |
| ^*^Predicted for parous women having their first birth at age 23, corresponding to median age at first birth in our study population. | | | | | | | | | | | | | | | | |  |
| ^†^ Estimates are based on the trajectory models depicted in Figure 2a and 2b and adjusted for age, HUNT survey, education and ever daily smoking. | | | | | | | | | | | | | | | | |  |

| **Supplemental Table 4. Mean within-woman change in systolic and diastolic blood pressure between HUNT2 and HUNT3 (n=1048).** | | | | | | | | | | |
| --- | --- | --- | --- | --- | --- | --- | --- | --- | --- | --- |
| **No. of births between HUNT2 and HUNT3** |  |  |  | **Systolic blood pressure (mmHg)** | | |  | **Diastolic blood pressure (mmHg)** | | |
|  |  | n |  | change^*^ | 95% CI | p-value |  | change^*^ | 95% CI | p-value |
| None |  | 426 |  | ref. |  |  |  | ref. |  |  |
| Any |  | 620 |  | -3.99 | [-5.98 , -1.99] | <0.001 |  | -3.04 | [-4.43 , -1.64] | <0.001 |
| 1 |  | 139 |  | -3.57 | [-6.20 , -0.94] | 0.008 |  | -3.05 | [-4.89 , -1.20] | 0.001 |
| 2 |  | 334 |  | -3.28 | [-5.53 , -1.04] | 0.004 |  | -2.53 | [-4.10 , -0.96] | 0.002 |
| ≥ 3 |  | 147 |  | -6.47 | [-9.26 , -3.68] | <0.001 |  | -4.34 | [-6.28 , -2.39] | <0.001 |
| ^*^estimates are adjusted for age and education at baseline (HUNT2) and change in smoking status from HUNT2 to HUNT3. | | | | | | | | | | |

| **Supplemental Table 5. Mean within-woman change in blood pressure (mmHg) due to pregnancy among 754^*^ women who participated in both HUNT2 and HUNT3 and had complete data on all covariates.** | | | | | | | | | | | | | | | | | | | | | | | | | |
| --- | --- | --- | --- | --- | --- | --- | --- | --- | --- | --- | --- | --- | --- | --- | --- | --- | --- | --- | --- | --- | --- | --- | --- | --- | --- |
| **No. of births between HUNT2 and HUNT3** | | |  |  | **Model 1**^†^ |  |  |  | **Model 2**^‡^ |  |  |  | **Model 3**^§^ | |  |  |  | **Model 4**^\|\|^ |  |  |  | **Model 5**^#^ |  | | |
|  |  |  |  | **change** | **95% CI** | **p-value** |  | **change** | **95% CI** | **p-value** |  | **change** | **95% CI** | **p-value** | |  | **change** | **95% CI** | **p-value** |  | **change** | **95% CI** | **p-value** | | |
| **Systolic (mmHg)** | | |  |  |  |  |  |  |  |  |  |  | | | |  |  |  |  |  |  | | |  |  |
| None (309) | | |  | ref. |  |  |  | ref. |  |  |  | ref. |  | |  |  | ref. |  |  |  | ref. |  |  | | |
| Any (431) | | |  | -3.25 | [-5.61, -0.89] | 0.007 |  | -3.07 | [-5.46, -0.69] | 0.012 |  | -3.13 | [-5.52, -0.74] | | 0.010 |  | -2.63 | [-5.06, -0.21] | 0.033 |  | -2.47 | [-4.86, -0.08] | 0.043 | | |
| 1 (92) | | |  | -2.62 | [-5.83, 0.59] | 0.110 |  | -2.54 | [-5.77, 0.68] | 0.123 |  | -2.62 | [-5.85, 0.62] | | 0.113 |  | -2.12 | [-5.38, 1.13] | 0.201 |  | -2.06 | [-5.28, 1.15] | 0.208 | | |
| 2 (226) | | |  | -2.63 | [-5.34, 0.07] | 0.056 |  | -2.48 | [-5.20, 0.24] | 0.073 |  | -2.52 | [-5.24, 0.20] | | 0.069 |  | -2.02 | [-4.77, 0.73] | 0.150 |  | -1.92 | [-4.63, 0.79] | 0.165 | | |
| ≥ 3 (113) | | |  | -5.39 | [-8.62, -2.16] | 0.001 |  | -5.15 | [-8.42, -1.87] | 0.002 |  | -5.22 | [-8.49, -1.94] | | 0.002 |  | -4.72 | [-8.01, -1.42] | 0.005 |  | -4.29 | [-7.55, -1.03] | 0.010 | | |
| **Diastolic (mmHg)** | | |  |  |  |  |  |  |  |  |  |  | | | |  |  |  |  |  |  |  |  | | |
| None (309) | | |  | ref. |  |  |  | ref. |  |  |  | ref. |  | |  |  | ref. |  |  |  | ref. |  |  | | |
| Any (431) | | |  | -2.33 | [-3.90, -0.75] | 0.004 |  | -2.37 | [-3.96, -0.78] | 0.004 |  | -2.37 | [-3.96, -0.77] | | 0.004 |  | -2.04 | [-3.66, -0.42] | 0.014 |  | -2.00 | [-3.62, -0.39] | 0.015 | | |
| 1 (92) | | |  | -2.17 | [-4.31, -0.03] | 0.047 |  | -2.26 | [-4.42, -0.11] | 0.040 |  | -2.21 | [-4.38, -0.05] | | 0.045 |  | -1.89 | [-4.07, 0.29] | 0.089 |  | -1.88 | [-4.05, 0.30] | 0.091 | | |
| 2 (226) | | |  | -1.83 | [-3.63, -0.02] | 0.047 |  | -1.86 | [-3.67, -0.04] | 0.045 |  | -1.86 | [-3.68, -0.05] | | 0.044 |  | -1.53 | [-3.37, 0.30] | 0.102 |  | -1.51 | [-3.35, 0.32] | 0.107 | | |
| ≥ 3 (113) | | |  | -3.60 | [-5.76, -1.45] | 0.001 |  | -3.69 | [-5.88, -1.50] | 0.001 |  | -3.72 | [-5.91, -1.53] | | 0.001 |  | -3.40 | [-5.61, -1.20] | 0.002 |  | -3.31 | [-5.51, -1.10] | 0.003 | | |
| ^*^ Sample size is lower than in Supplemental Table 3 predominantly due to incomplete information on oral contraceptive use from questionnaires retrieved by mail.  ^†^estimates are adjusted for age at baseline (HUNT2). | | | | | | | | | | | | | | | | | | | | | | | |  |  |
| ^‡^ estimates are adjusted for age and education at baseline (HUNT2). | | | | | | | | | | | | |  | |  |  |  |  |  |  |  |  |  |  |  |
| ^§^ estimates are adjusted for age and education at baseline (HUNT2) and change in smoking status from HUNT2 to HUNT3. | | | | | | | | | | | | | | | | | |  |  |  |  |  |  |  |  |
| ^\|\|^ estimates are adjusted for age and education at baseline (HUNT2) and change in smoking status and oral contraceptive use from HUNT2 to HUNT3. | | | | | | | | | | | | | | | | | | |  |  |  |  |  |  |  |
| ^#^ estimates are adjusted for age and education at baseline (HUNT2) and change in smoking status, oral contraceptive use and BMI from HUNT2 to HUNT3. | | | | | | | | | | | | | | | | | | |  |  |  |  |  |  |  |
|  |  |  |  |  |  |  |  |  |  |  |  |  |  |  |  |  |  |  |  |  |  |  |  |  |  |

| **Supplemental Table 6. Mean within-woman change in systolic and diastolic blood pressure between HUNT2 and HUNT3 by breastfeeding categories (n=702).**^*^ | | | | | | | | | |
| --- | --- | --- | --- | --- | --- | --- | --- | --- | --- |
|  |  |  | **Systolic blood pressure (mmHg)** | | |  | **Diastolic blood pressure (mmHg)** | | |
| **Breastfeeding status of first pregnancy** | N |  | change^†^ | 95% CI | p-value |  | change^†^ | 95% CI | p-value |
| No pregnancy | 320 |  | reference |  |  |  | reference |  |  |
| No breastfeeding | 15 |  | -2.89 | [-9.90 , 4.13] | 0.420 |  | -2.85 | [-7.60 , 1.90] | 0.239 |
| > 0 to < 3 months | 26 |  | -2.35 | [-7.80 , 3.09] | 0.397 |  | -2.72 | [-6.40 , 0.97] | 0.149 |
| 3 to 6 months | 41 |  | -1.42 | [-5.93 , 3.10] | 0.539 |  | -1.11 | [-4.17 , 1.95] | 0.476 |
| > 6 months | 300 |  | -2.71 | [-5.32 , -0.10] | 0.042 |  | -2.46 | [-4.23 , -0.70] | 0.006 |
| ^*^sample is smaller than in Supplemental Table 4 due to missing information on breastfeeding length.  ^†^estimates are adjusted for age and education at baseline (HUNT2) and change in smoking status, oral contraceptive use and BMI from HUNT2 to HUNT3. | | | | | | | | | |


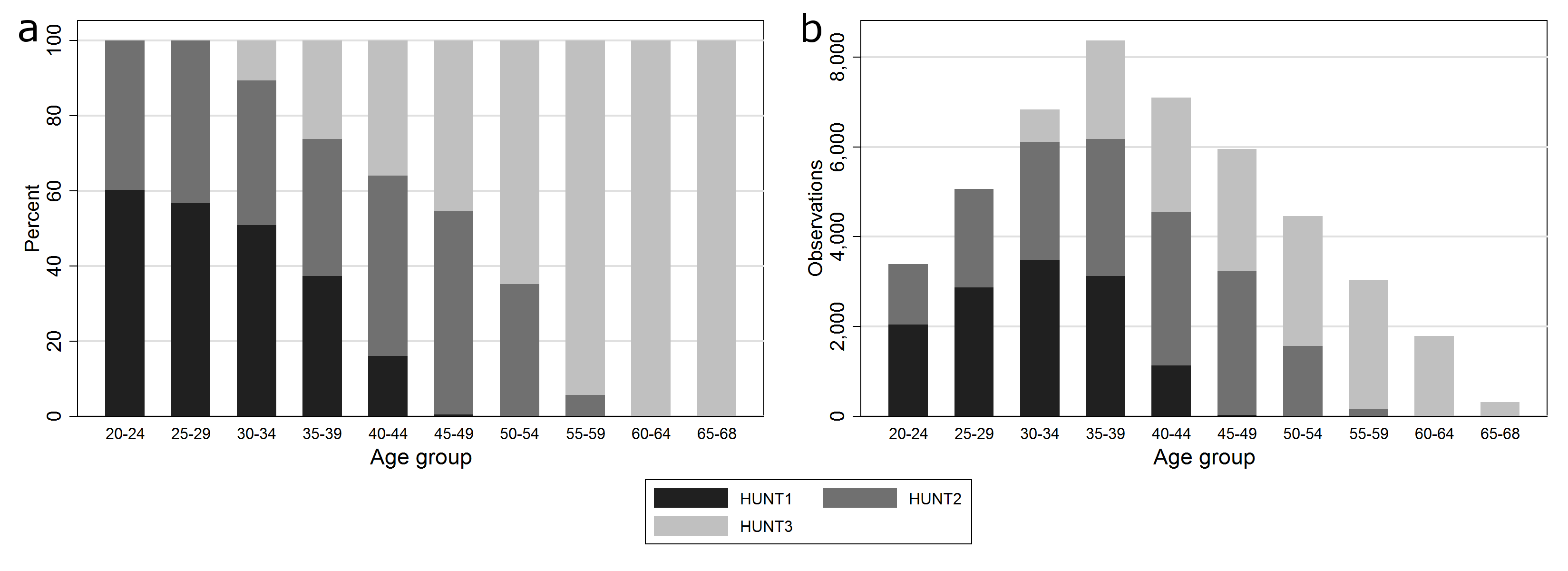


**Supplemental Figure 1**. **Proportion (a) and number (b) of blood pressure measurements according to age at participation and HUNT survey.**


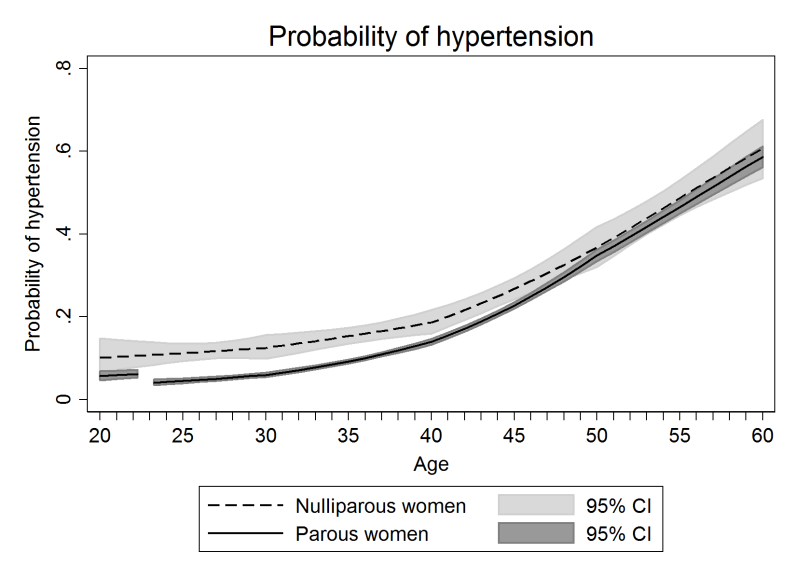


**Supplemental Figure 2. Predicted probability of hypertension by age for nulliparous and parous women.** The gap in the graph for parous women corresponds to the 1^st^ pregnancy and 3-month postpartum period with the 1^st^ birth at age 23. Estimates are adjusted for age, HUNT survey, education and ever daily smoking.


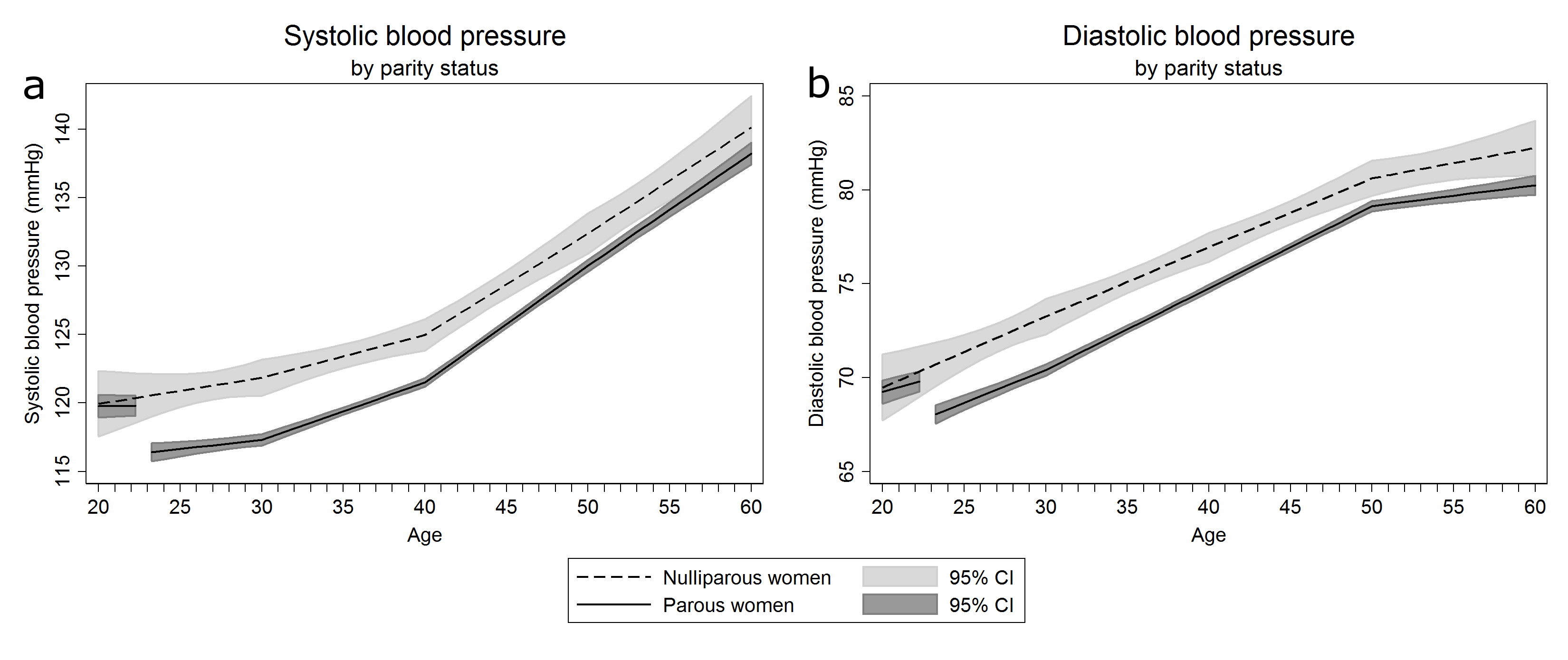


**Supplemental Figure 3**. **Mean systolic and diastolic blood pressure life course trajectories for nulliparous and parous women.** This strictly longitudinal sensitivity analysis includes only women with at least two blood pressure observations (n=15,233). Trajectories are drawn for women with covariates fixed at their means and with gaps in the graphs of parous women corresponding to the 1^st^ pregnancy and 3-month postpartum period with the 1^st^ birth at age 23. Estimates are adjusted for age, HUNT survey, education and ever daily smoking.


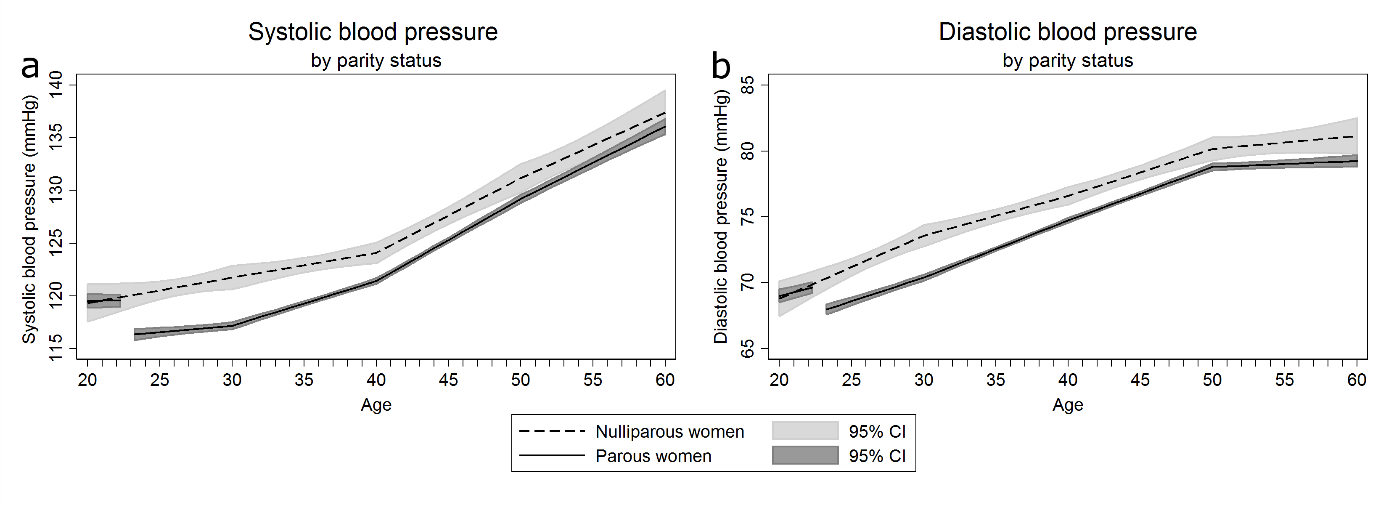


**Supplemental Figure 4**. **Mean systolic (a) and diastolic (b) blood pressure life course trajectories for nulliparous and parous women using original blood pressure data.** This sensitivity analysis is based on the original blood pressure values without adding constants in women who used antihypertensive medication, as was done in the main analysis. Trajectories are drawn for women with covariates fixed at their means and with gaps in the graphs of parous women corresponding to the 1^st^ pregnancy and 3-month postpartum period with the 1^st^ birth at age 23. Estimates are adjusted for age, HUNT survey, education and ever daily smoking.


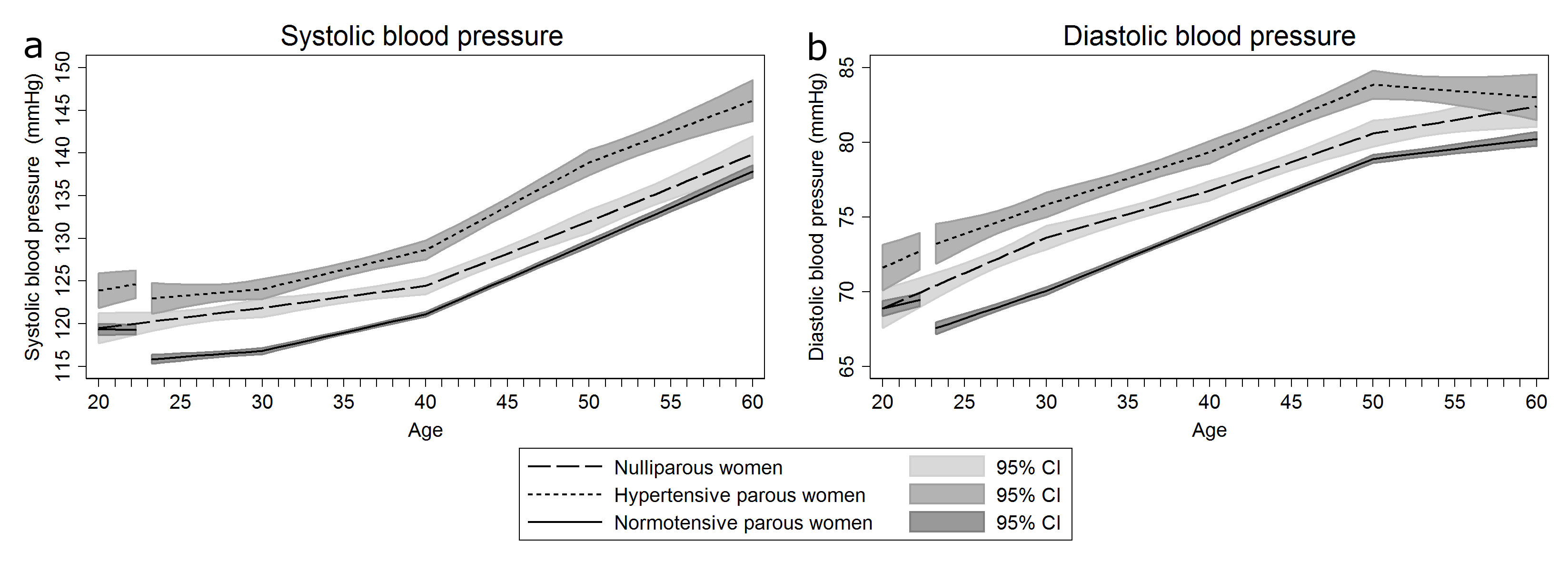


**Supplemental Figure 5**. **Mean systolic (a) and diastolic (b) blood pressure life course trajectories for nulliparous women and parous women with or without a hypertensive disorder in their first pregnancy.** Trajectories are drawn for women with covariates fixed at their means and with gaps in the graphs of parous women corresponding to the 1^st^ pregnancy and 3-month postpartum period with the 1^st^ birth at age 23. Estimates are adjusted for age, HUNT survey, education and ever daily smoking.
